# Ultrasound-guided minimally invasive thoracotomy for removal of migrating grass Awn in a Cat

**DOI:** 10.1007/s11259-026-11072-z

**Published:** 2026-01-20

**Authors:** Vicente Francisco Ratto Valderrama, Federica Valeri, Domenico Caivano, Giulia Moretti, Antonello Bufalari

**Affiliations:** Department of Veterinary Medicine, University of Perugia, Via San Costanzo 4, 06126 Perugia, Italy

**Keywords:** Foreign body, Foxtail, Surgery, Ultrasonography, Feline

## Abstract

Migrating grass awns are a rare cause of thoracic reactions in cats, resulting in pleural effusion, inflammation, infection and granulomatous responses. Diagnosis and localization are challenging, and treatment commonly requires invasive surgical intervention. We report a case of a 5-year-old, neutered male, domestic shorthair cat referred for pleural effusion and suspected intrathoracic vegetal foreign body. Thoracic ultrasonography confirmed the presence of pleural effusion and a linear hyperechoic structure within the pleural space was visualized. An ultrasound-guided minimally invasive thoracotomy was performed through the right fifth intercostal space and intraoperative ultrasonography was useful to precisely locate and remove the foreign body using a Hartmann ear forceps. Cytological analysis was performed on the pleural fluid and bacteriological analysis was performed on both the pleural fluid and the retrieved foreign body. The cytologic findings were consistent with an exudative effusion but no bacteria were isolated on both direct and enrichment bacterial cultures; empirical antibiotic treatment with marbofloxacin was continued. The patient recovered uneventfully, and follow-up revealed complete resolution of the clinical signs. This case report highlights the effectiveness of ultrasound-guided minimally invasive approach for intrathoracic vegetal foreign body removal in a cat. The transthoracic and intraoperative ultrasound can be useful for visualization and exact localization of the grass awn, minimizing surgical trauma and improving the outcome.

## Background

Barbed vegetal foreign bodies can enter the body through inhalation or transcutaneous penetration, especially in outdoor animals; whereas this is well recognized in hunting or working dogs, it remains less frequently reported in cats (Schultz and Zwingenberger 2008; Cola et al. 2019; Caivano et al. 2023). Feline behavior, including frequent grooming, the predominance of closed-mouth breathing, and a generally low likelihood of ingesting foreign objects, makes vegetal foreign bodies relatively rare in this species. Nonetheless, cats that spend time outdoors can still come into contact with plant material capable of penetrating the respiratory tract. (Cola et al. 2019).

Once inside, these awns are able to migrate through tissues in a unidirectional manner: the sharp anterior end allows penetration, while the backward-pointing barbs anchor the awn to surrounding tissues and prevent retrograde movement or spontaneous expulsion. It is previously described that inhaled grass awns can reach the lower airways via the trachea and caudal bronchi, eventually perforating lung tissue and migrating into adjacent structures such as the pleural or pericardial space or even the abdominal and retroperitoneal cavities (Frendin et al. 1994, 1999; Caivano et al. 2014, 2016, 2019; Birettoni et al. 2017; Hopper et al. 2004; Marchesi et al. 2020). This leads to progressive tissue disruption and can result in chronic inflammation, granuloma formation, abscessation, or septic pleuritis. In some cases, clinical signs can be subtle or transient. This is particularly true when they are partially masked by empirical treatment with antibiotic and/or anti-inflammatory drugs. This can delay diagnosis and allow the migration of the grass awn through the body.

Diagnosis is often challenging and imaging modalities such as ultrasonography, CT, or MRI can aid in the initial diagnosis (Schultz and Zwingenberger 2008; Swinbourne et al., 2011; Whitty et al., 2013; Caivano et al. 2023). Ultrasonography has been previously reported to detect, localize, and guide removal of vegetal foreign bodies in dogs and cats (Caivano et al. 2016; Cola et al. 2019). However, ultrasonography can fail to detect the vegetal foreign body especially if the migration occurs in the pleural space: the presence of scattered hyperechoic elements (fibrin, tissue remnants, cellular debris, or air) can limit visualization of the awn (Caivano et al. 2016). Definitive treatment requires surgical removal of the foreign body. Traditional thoracotomy is effective, but can be associated with complications and morbidity (tissue trauma, postoperative pain, and long recovery time) (Stillion and Letendre 2015, Cola et al. 2019). Minimally invasive techniques, such as thoracoscopy or targeted thoracotomy under imaging guidance, offers a less traumatic alternative (Pelàez and Jolliffe 2012, Caivano et al. 2014).

This case report aims to describe the successful removal of a migrating intrathoracic grass awn in a cat using an ultrasound-guided minimally invasive approach.

## Case presentation

A five-year-old, neutered male domestic shorthair, was referred to the Veterinary Teaching Hospital of Perugia University for pleural effusion and suspected intrathoracic vegetal foreign body. Thoracic radiographs performed by the referring veterinarian showed moderate pleural effusion with loss of cardiac and diaphragmatic silhouette definition. The referring veterinarian had visualized a hyperechoic structure during the ultrasonographic examination of the thorax. The cat was treated with marbofloxacin (2 mg/kg, PO, q 24 h) for two days prior to presentation.

**Fig. 1:**
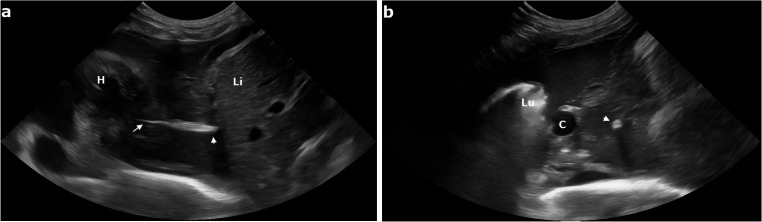
Long axis (**a**) and short axis (**b**) transthoracic ultrasonographic image of the caudal mediastinum showing pleural effusion and a spindle-shaped hyperechoic foreign body consistent with a grass awn. The tip (arrowhead) and the barbs (arrow) of the awn are evident in long axis image (a). The vegetal foreign body (arrowhead) shows an acoustic shadowing in short axis (b). H, heart; Li, liver; Lu, lung; C, caudal vena cava

On physical examination, the cat revealed dyspnea and hyperthermia (39.5 °C). Thoracic ultrasonography was performed using a Samsung V8 system (Samsung Healthcare Italia, Milan, Italy) equipped with a 4–10 MHz microconvex probe. The probe was placed sequentially along each intercostal space following a systematic scanning protocol. The examination confirmed the presence of pleural effusion, appearing moderately echogenic and heterogenous, consistent with a purulent exudate. A hyperechoic, linear structure located within the right caudal mediastinum was also visualized. This structure was characterized by two hyperechogenic bands casting an acoustic shadow, consistent with a migrating vegetal foreign body (Fig. 1).

Based on these findings, surgical removal of the migrating grass awn was planned. Preoperative ultrasonographic examination repeated under general anesthesia on the day of the surgery, revealed cranial migration of the vegetal foreign body. It was now visualized within the cranial mediastinum near the heart. Since the grass awn was single, well visualized on ultrasound, the decision was made to remove the foreign body percutaneously under real-time ultrasonographic guidance as first attempt.

The cat was premedicated with methadone (0.2 mg/kg, IV), and 15 min later, a 22-gauge catheter was aseptically placed in the right cephalic vein. Supplemental oxygen was provided for 10 min via face mask prior to the induction of anesthesia with propofol (3 mg/kg, IV to effect) until an adequate depth of anesthesia was achieved. Prior to endotracheal intubation, lidocaine (1%) injectable solution (0.3 mg/kg) was instilled topically on the arytenoid cartilages via a syringe and a 20-gauge IV catheter. The patient was then orotracheally intubated with a cuffed Murphy endotracheal tube (internal diameter, 3.0 mm) and connected to a rebreathing circuit Y, at a rate of 2 L/min. Anesthesia was maintained with delivery of isoflurane in oxygen during manually controlled ventilation. Monitoring consisted of continuous electrocardiography, indirect measurement of blood pressure, and pulse oximetry, end-tidal partial pressure of carbon dioxide (measured by means of capnography), and end-tidal isoflurane concentration were monitored continuously with a multiparameter monitor and recorded every 5 min. Lactated Ringer solution (10 mL/kg/h) was administered IV during anesthesia.

**Fig. 2:**
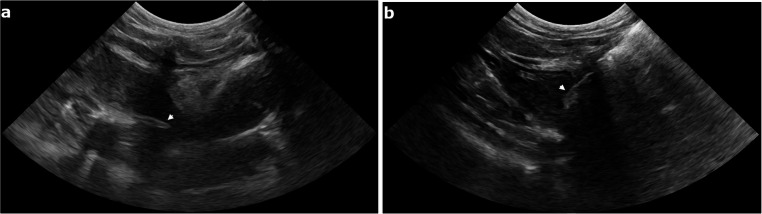
Intraoperative ultrasonographic image of the grass awn (arrowhead) migrated within the cranial mediastinum (**a**) and the Hartmann ear forceps (arrowhead) advanced through the thoracic incision within the pleural space (**b**)

**Fig. 3:**
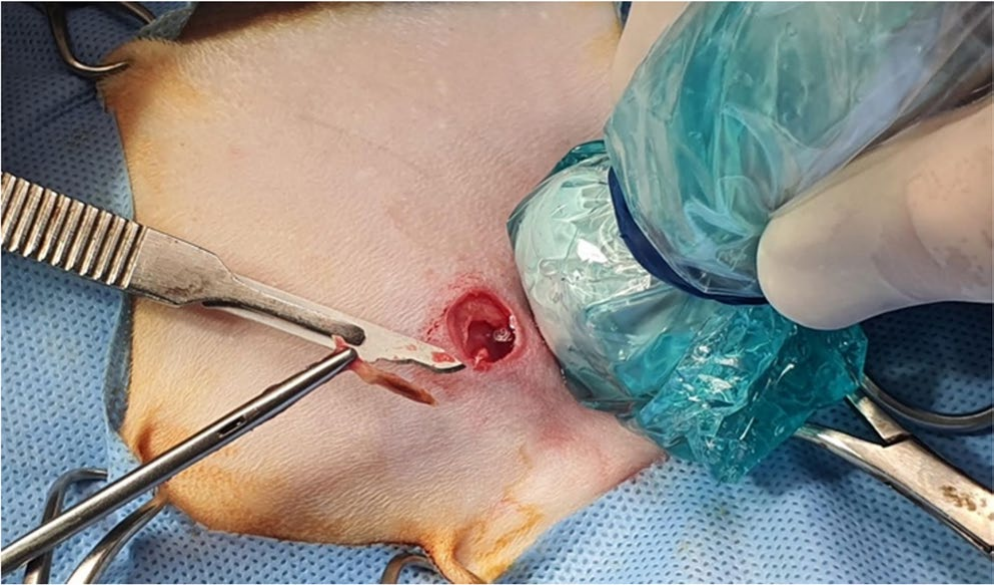
Intraoperative photograph illustrating the surgical field and successful extraction of the vegetal foreign body. The image shows the small thoracotomy site (approximately 10 mm x 10 mm) over the right cranial hemithorax, the Hartmann ear forceps holding the retrieved grass awn, a #15 scalpel blade placed alongside for scale reference and the ultrasound probe used for echo-guided procedure

The patient was positioned in left lateral recumbency. Trichotomy and aseptic preparation were performed over the right hemithorax. A small square skin incision (10 mm in length x 10 mm in width) was made at the level of the 5th intercostal space, just ventral to the costochondral junction. The underlying pectoral muscle was gently retracted to expose the intercostal musculature. A blunt incision of the external and internal intercostal muscles (about 5 mm in depth) was performed to easily establish a point of egress for a small blunt probe, which was used to precisely localize the foreign body. Under continuous ultrasonographic guidance, the probe was positioned tangential to the heart in direct contact with the tip of the grass awn, allowing optimal alignment for the surgical approach. Once the foreign body was precisely localized, the guide probe was withdrawn and replaced with a Hartmann ear forceps. The forceps was advanced through the thoracic incision, and the vegetal foreign body was carefully grasped and extracted in a single maneuver under real-time ultrasound guidance (Figs. 2 and 3).

Prior to closure, approximately 40 mL of moderately turbid, purulent fluid was aspirated from the pleural cavity under direct ultrasonographic guidance using a small-gauge catheter inserted through the right fifth intercostal space. Before standard fascial and skin closure, a 12 French (4 mm) chest drain was placed under continuous ultrasonographic guidance, allowing real-time visualization of the pleural space and accurate positioning. Post-placement radiographs were also obtained to confirm chest drain position. The cat received postoperative buprenorphine to manage pain associated with the drain. Pleural lavage (using 25 ml/kg of warmed sterile saline 0,9% solution) was performed immediately after placement of the thoracic drainage tube and repeated daily for two days. No intra-operative complication occurred during the procedure. No lung injuries occurred since no pleural adhesions were encountered when the internal intercostal muscle was incised. Neither blunt probe inspection nor grasping forceps retrieval caused any damage to the internal thoracic organs. The operative time, measured from skin incision to removal of the vegetal foreign body, was approximately 15 min. The retrieved foreign body, consistent with a grass awn (*Hordeum* spp.) based on gross morphological characteristics, and the aspirated pleural effusion were submitted for microbiological culture, both yielding negative results, including after enrichment culture. Cytological analysis of the pleural fluid, evaluated on four cytospin-prepared slides, revealed a mixed inflammatory cell population with a predominance of mature segmented neutrophils (50%), some exhibiting degenerative changes. Activated macrophages accounted for 30% of the cells, and lymphocytes for 20%. Occasional reactive mesothelial cells were observed, and no microorganisms were identified. The cytologic findings were consistent with a sterile pyogranulomatous inflammation secondary to a foreign body. Despite the absence of identifiable bacteria, the presence of degenerate neutrophils supported the decision to maintain empirical antibiotic treatment (marbofloxacin).

A thoracic bandage was maintained for three days postoperatively because of the nature of the disease (pyothorax), and the chest drain remained in place during this period, being removed once no further fluid or air was observed from the thoracic cavity. The patient was discharged three days after surgery, and sutures were scheduled for removal ten to twelve days following the procedure. A thoracic ultrasound was performed prior to discharge, confirming resolution of the effusion and the absence of immediate postoperative complications. A follow-up thoracic ultrasound was also planned at the conclusion of antibiotic therapy (after 10 days) to confirm continued resolution and to rule out any residual or recurrent clinical signs. Long-term follow-up via clinical examinations and owner reports over the subsequent 2 years showed no recurrence of clinical signs, and the patient remains healthy.

## Discussion and conclusions

Migration of vegetal foreign bodies, such as *Hordeum* spp. grass awns, is relatively uncommon in cats and it can represent a significant diagnostic and therapeutic challenge due to their subtle clinical presentation and complexity of the detection and removal. Cola et al. described the use of ultrasonography in detecting thoracic grass awns in 4 cats presented for respiratory signs (Cola et al. 2019). Ultrasound examination showed unilateral pleural effusion and atelectasis of the ventral portion of the lung lobes of the affected side in all cats. In one cat, a mediastinal spindle-shaped structure characterized by two hyperechogenic walls separated by a central hypoechogenic band, compatible with a vegetal foreign body, was visualized during the first ultrasonographic scan. In the other 3 cats, 3 to 5 ultrasonographic examinations repeated on different days were required to identify the foreign bodies in the thoracic cavity. In contrast, in the present case, the grass awn was successfully identified on the first ultrasound examination, highlighting that immediate detection is possible when the foreign body is well localized. This lower incidence in cats compared to dogs can be related to distinct feline behaviors, such as meticulous grooming, predominantly nasal breathing with the mouth closed, and a reduced tendency to ingest foreign material. These explanations remain hypothetical, as direct evidence supporting them is limited (Schultz et al. 2008; Koutinas et al. 2003; Leal et al. 2017). When vegetal foreign bodies migrate into the thoracic cavity, their visualization can be particularly challenging. Pulmonary consolidation associated with migrating grass awn can help as an indirect indicator, guiding the ultrasonographer toward the area of interest and facilitating localization of the migrating foreign body (Caivano et al. 2023). Ultrasonographic visualization of the vegetal foreign body within the pleural space is often limited by the presence of hyperechoic artifacts such as fibrin, tissue fragments, cellular debris, or small air bubbles. In our case, the vegetal foreign body was visualized within the pleural space and ultrasonography revealed its spontaneous movements within the effusion and migration, facilitating the localization and planning the surgical approach. Visualization of the grass awn was feasible likely due to the moderate echogenicity of the effusion, its favorable location, and the minimal presence of fibrin, which together enhanced image quality.

The standard surgical approach for removing migrating grass awns in the thoracic cavity typically involves either thoracotomy or thoracoscopy in dogs and cats. Thoracotomy, whereas providing direct access and visibility, is associated with tissue trauma, postoperative pain, and longer hospitalization and recovery times. Thoracoscopy, despite being less invasive and associated with faster recovery, requires specialized equipment and advanced technical skills. Both thoracotomy and thoracoscopy carry some risk of complications; however, hospital length of stay has not been shown to differ significantly between patients treated with both procedures (Stillion and Letendre 2015). Moreover, thoracoscopy cannot always be a feasible option: chronic inflammation, often associated with long-standing foreign body presence, can lead to the formation of pleural adhesions. These adhesions impair visualization and limit the maneuverability of thoracoscopic instruments, reducing the feasibility of a thoracoscopic approach. In such challenging settings, real-time ultrasonographic guidance can be especially valuable: although it does not offer the same direct visualization as thoracoscopy, it allows precise localization of mobile or difficult-to-reach foreign bodies and could be a less-invasive alternative when thoracoscopy is unavailable. However, ultrasound has intrinsic limitations: small, discrete grass awns or those located in less accessible areas can be missed with a percutaneous approach and the technique cannot be suitable in cases with poor ultrasonographic visibility or the presence of pleural adhesions. In such cases, conversion to open-chest surgery (thoracotomy) is often necessary to ensure safe and complete foreign body removal. Cola et al. reported the surgical approach in 4 cats with migrating intrathoracic grass awns. In 2 cats the surgical approach consisted of a video-assisted thoracoscopy with the cat in dorsal recumbency. However, in 1 of these cats video-assisted thoracoscopy required conversion to intercostal thoracotomy because of numerous adhesions between the visceral and parietal pleura. In another 2 cats, intercostal thoracotomy was used to reach the mediastinum. In all cats, the vegetal foreign body was removed. In our case, the combination of real-time ultrasonographic guidance with a minimally invasive surgical technique allowed for successful removal of the grass awn. Moreover, the precise localization of the mobile foreign body allowed us to minimize surgical trauma and increase procedural safety. However, even with ultrasound visualization of the foreign body, remains a risk of inadvertently grasping surrounding tissue while manipulating instruments. Finally, the cat was discharged just three days postoperatively with minimal surgical morbidity and a rapid recovery.

Cytological analysis of the pleural fluid revealed a purulent exudate with no visible microorganisms and negative results on both direct and enrichment bacterial cultures. This is not surprising, considering that the cat had received marbofloxacin prior to referral and could have been influenced by prior treatment. This preexisting antimicrobial therapy can have suppressed bacterial growth or eliminated susceptible pathogens, potentially masking an underlying infection. Nevertheless, a sterile pyogranulomatous reaction secondary to the presence of a foreign body cannot be excluded.

In conclusion, this case highlights the diagnostic and therapeutic value of the ultrasonography for managing migrating intrathoracic vegetal foreign bodies in a feline patient and suggests that, in selected cases, a tailored minimally invasive approach could provide an effective alternative to more invasive traditional techniques. Ultrasonography, guiding entirely the procedure, facilitated localization and reduced surgical trauma, potentially leading to faster recovery compared to more invasive approaches. This ultrasound-guided percutaneous approach can be suitable for cases with a well-localized, mobile foreign body in the thoracic cavity and minimal pleural adhesions or chronic inflammation, whereas cases with multiple foreign bodies, extensive adhesions, or diffuse pleural disease can require thoracotomy or thoracoscopy. In the present case, the foreign body was distinctly localized and accessible, making it an ideal candidate for this minimally invasive approach. Although intrathoracic migration of vegetal foreign bodies is rarely reported in feline patients, it remains a critical differential diagnosis in cases of pleural effusion.

## Data Availability

The data presented in this study are available in the article.
